# Epidemiologic Trends and Distributions of Imported Infectious Diseases Among Travelers to Japan Before and During the COVID-19 Pandemic, 2016 to 2021: A Descriptive Study

**DOI:** 10.2188/jea.JE20230025

**Published:** 2024-04-05

**Authors:** Ayu Kasamatsu, Kazuhiko Kanou, Munehisa Fukusumi, Yuzo Arima, Shun Omori, Haruna Nakamura, Tetsuro Sato, Yusuke Serizawa, Asuka Takeda, Hiroyuki Fujikura, Chiaki Ikenoue, Shingo Nishiki, Yoshihiro Fujiya, Takeshi Arashiro, Takuri Takahashi, Tomoe Shimada, Motoi Suzuki, Tomimasa Sunagawa

**Affiliations:** 1 Field Epidemiology Training Program, National Institute of Infectious Diseases, Tokyo, Japan; 2 National Institute of Infectious Diseases, Tokyo, Japan

**Keywords:** COVID-19, epidemiology, imported infectious disease, surveillance, travel

## Abstract

**Background:**

Little is known about the trends of imported infectious diseases among travelers to non-endemic countries during the novel coronavirus disease 2019 (COVID-19) pandemic. This article aimed to describe those among travelers to Japan.

**Methods:**

This is a descriptive study based on national surveillance data. Imported infectious disease cases were defined as those with a reported overseas source of infection among 15 diseases pre-selected based on the probability and impact of importation. The number of notified cases from April 2016 to March 2021 were described by disease and time of diagnosis. The relative ratio and absolute difference in case counts—both by number and per arrival—were calculated by disease comparing those from the pandemic period (April 2020–March 2021) to the pre-pandemic period (April 2016–March 2020).

**Results:**

A total of 3,524 imported infectious disease cases were diagnosed during the study period, including 3,439 cases before and 85 cases during the pandemic. The proportionate distribution of diseases changed but notification counts of all 15 diseases decreased during the pandemic. Accounting for arrivals, however, seven diseases showed a two-fold or greater increase, with a notable absolute increase per million arrivals for amebiasis (60.1; 95% confidence interval [CI], 41.5–78.7), malaria (21.7; 95% CI, 10.5–33.0), and typhoid fever (9.3; 95% CI, 1.9–16.8).

**Conclusion:**

The epidemiology of imported infectious diseases changed during the pandemic. While the number of imported infectious disease cases decreased, the number of cases per arrivals increased considerably both in relative and absolute terms for several diseases of public health and clinical importance.

## INTRODUCTION

In response to novel coronavirus disease 2019 (COVID-19), which was declared a pandemic by the World Health Organization on March 11, 2020,^1^ governments globally implemented international travel restrictions.^2^ These measures have been followed by a substantial decline in the number of international travelers worldwide.^2^ Japan has been no exception. Since February 2020, Japan had taken measures to enhance border control measures, including entry restrictions depending on the country of departure for foreign nationals.^3^ Subsequently, the total number of travelers entering Japan in 2020 fell sharply from the hitherto upward trend.^4^ On the other hand, some exceptions were allowed, such as permitting foreign nationals with “special exceptional circumstances” (eg, spouses or children of Japanese nationals/permanent residents, or foreign nationals with residency status of “Diplomat” or “Official”) to enter Japan,^3^ and establishing special quota pertaining to cross-border business travelers between Japan and certain countries and regions.^5^ In this context, not only the number of travelers to Japan but also traveler characteristics and countries of travel origin could have changed during the pandemic.

Several studies have shown that, since before the pandemic, air travel data, such as the number and demographic characteristics of travelers as well as their countries of origin, are associated with patterns of infectious disease importations.^6^^–^^9^ Given the situation in Japan under the COVID-19 pandemic, it is possible that the number and distributions of infectious disease cases among travelers to non-endemic countries had changed. The aim of this study was to describe the important changes in trends and distributions of imported infectious diseases among travelers entering Japan before and during the pandemic.

## METHODS

### Study design

This is a retrospective, mostly descriptive analysis of national surveillance data combined with publicly available national migration statistics.

### Data sources

The National Epidemiological Surveillance of Infectious Diseases (NESID) system has been operating under the Infectious Diseases Control Law since 1999. Physicians are required to notify all notifiable diseases to the public health centers (the reporting criteria and the notification form for each disease are publicly available),^10^ who coordinate with prefectural and municipal public health institutes (eg, laboratory testing). The notification form includes demographic, clinical, laboratory, and exposure information. The data collected are then reported by the public health centers and institutes via the electronic NESID system. We extracted data on cases diagnosed between April 1, 2016 and March 31, 2021 on December 28, 2021. The variables used in the analysis were patient name (to differentiate between Japanese and non-Japanese), sex, symptoms/signs, diagnostic methods, date of diagnosis, presumed date of infection, and suspected country of infection.

The monthly number of arrivals into Japan and their nationality was obtained in April 2022 from the Immigration Services Agency of Japan website.^4^ Demographic and travel-related information of arrivals, including length of stay and status of residence, were obtained from the annual report on the same website.

### Case definition

In 2018, 15 notifiable diseases with a historically sizable number and proportion of imported cases were systematically selected as priority imported infectious diseases by the National Institute of Infectious Diseases; these 15 have been monitored continuously since then. These are amebiasis, chikungunya, cryptosporidiosis, dengue, giardiasis, hepatitis A, hepatitis E, leptospirosis, malaria, measles, paratyphoid fever, rubella, shigellosis, typhoid fever, and Zika virus disease. In accordance with the definition, an “imported case” was defined as a reported case whose source of infection was determined to be overseas by the physician who diagnosed and reported the case.

### Data analysis

We described the number of arrivals in Japan by month and by fiscal year (FY) running from April through March. Demographic and travel-related information were presented by calendar year owing to the limitations of the available data.

We described the monthly trends in the number of 15 imported infectious disease cases and the annual trends in the proportionate distribution of the 15 diseases. We then assessed the importation data by first describing the case counts before (April 1, 2016 to March 31, 2020, corresponding to FY 2016–2019) and during the pandemic period (April 1, 2020 to March 31, 2021, corresponding to FY 2020), based on the timing of the sharp decline in the number of travelers due to travel restrictions imposed by the government.^4^ Next, we compared the case counts by calculating the ratio and the difference between the two periods; the ratio provides a relative comparison while the difference indicates an absolute change. To account for the denominator of number of travelers, we similarly calculated the ratios and differences, per million arrivals, for the respective periods using the aggregate number of arrivals of 184,042,453 in FY 2016–2019 and 697,618 in FY 2020.^4^ The 95% confidence interval (CI) for the ratio and difference per million arrivals before and during the pandemic was estimated to indicate the level of precision.

Amebiasis and malaria, with the largest absolute increase in the number of cases per arrivals, were further analyzed to explore the potential reasons for this increase. We described amebiasis cases based on sex, symptoms, and FY of infection. Amebiasis cases with specific colonic mucosal lesions or positive fecal occult blood but that were otherwise asymptomatic were categorized as asymptomatic cases.^11^ The ratio and the difference of non-Japanese malaria cases per million foreign national arrivals, before and during the pandemic, were described by region, based on the travel origin of the cases and nationality of the arrivals. Japanese nationals were excluded from the analysis because of a lack of regional denominator data. Cases whose suspected country of infection were unknown or included more than one country were excluded from the analysis by region. Statistical analysis was performed using Stata/MP version 16.0 (Stata Corp, College Station, TX, USA).

### Ethical consideration

Information on notified cases was collected under the Infectious Diseases Control Law. The use of national surveillance data for public health purposes does not require informed consent from the patient or ethical approval from the relevant authorities. For those diseases that included identifiable data, strict data management practices were implemented per standard protocol.

## RESULTS

### Arrivals to Japan

**Figure 1.**
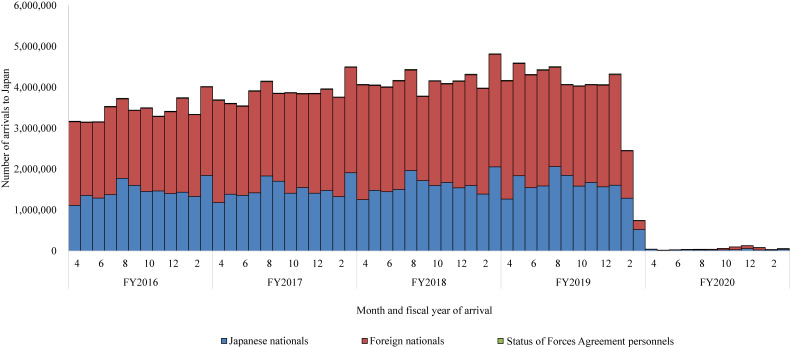
Number of arrivals to Japan by month of arrival, by nationality, April 2016–March 2021

The traveler volume to Japan dropped remarkably during February–April 2020 and remained low from April 2020 onwards (Figure 1). The total annual number of arrivals in FY 2020 was 697,618, a 98% decrease from the annual average of 46,641,690 in FY 2016–2019 (Table 1). While the proportion of foreign national arrivals exceeded that of Japanese nationals in FY 2016–2019, this was not the case in FY 2020. Asian nationals consistently constituted the majority of foreign national arrivals across all 5 years, and the distribution of nationalities from other regions remained largely stable. The proportion of African nationals remained low, averaging 0.2% in FY 2016–2019 and 1.2% in FY 2020.

**Table 1.** Number of arrivals to Japan, April 2016–March 2021

		FY 2016		FY 2017		FY 2018		FY 2019		FY 2020	
Total arrivals		41,529,240		46,603,677		50,096,935		45,812,601		697,618	
Japanese		17,455,991	42.0%	17,981,203	38.6%	19,237,954	38.4%	18,403,845	40.2%	348,741	50.0%
Non-Japanese		23,904,199	57.6%	28,443,905	61.0%	30,666,253	61.2%	27,223,118	59.4%	323,742	46.4%
Region of nationality	Africa	38,042	(0.2%)	40,181	(0.1%)	44,001	(0.1%)	56,858	(0.2%)	3,837	(1.2%)
	Asia	20,072,119	(84.0%)	24,170,161	(85.0%)	25,974,815	(84.7%)	22,328,118	(82.0%)	274,896	(84.9%)
	Europe	1,506,059	(6.3%)	1,665,549	(5.9%)	1,831,814	(6.0%)	1,957,879	(7.2%)	19,140	(5.9%)
	North America	1,642,910	(6.9%)	1,825,944	(6.4%)	2,014,017	(6.6%)	2,025,215	(7.4%)	13,332	(4.1%)
	Oceania	513,686	(2.1%)	588,693	(2.1%)	639,750	(2.1%)	690,584	(2.5%)	2,164	(0.7%)
	South America	130,163	(0.5%)	152,519	(0.5%)	161,025	(0.5%)	163,634	(0.6%)	10,343	(3.2%)
	No nationality	1,220	(0.0%)	858	(0.0%)	831	(0.0%)	830	(0.0%)	30	(0.0%)
SOFA personnel		169,050	0.4%	178,569	0.4%	192,728	0.4%	185,638	0.4%	25,135	3.6%

FY, fiscal year (from April to March of the subsequent year); SOFA, Status of Forces Agreement.

% represents the proportion among total arrivals for the fiscal year (% in parentheses represent the proportion among non-Japanese [ie, foreign] nationals).

The demographic and travel-related statistics of arrivals by nationality are presented in eFigures 1 and 2. The proportion of Japanese national arrivals who stayed at their travel origin for more than a month increased noticeably during the pandemic, compared to the pre-pandemic period (eFigure 1). During the pandemic, the proportion of temporary foreign visitors decreased, while the proportion of arrivals with employment qualifications or residency status increased (eFigure 2).

### Notification trends and distributions of 15 imported infectious diseases

**Figure 2.**
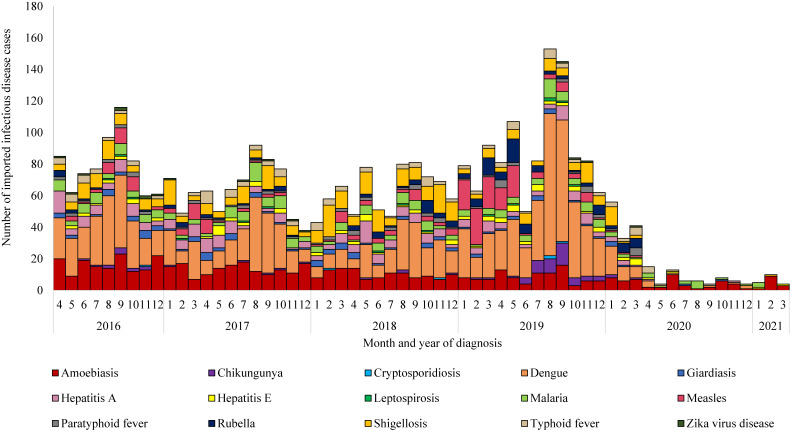
Number of imported infectious disease cases by month of diagnosis, by disease, Japan, April 2016–March 2021 (restricted to pre-selected 15 priority notifiable diseases)

A total of 3,524 imported infectious disease cases were diagnosed during FY 2016–2020, including 3,439 cases during FY 2016–2019 and 85 cases in FY 2020. During the pandemic FY 2020 period, 8/15 diseases were reported but chikungunya, cryptosporidiosis, leptospirosis, measles, paratyphoid fever, rubella, and zika virus infection were not reported. The monthly number of reported cases declined over February–April 2020 and remained low thereafter (Figure 2). The decline in case counts coincided with the drop in the number of travelers.^4^

**Figure 3.**
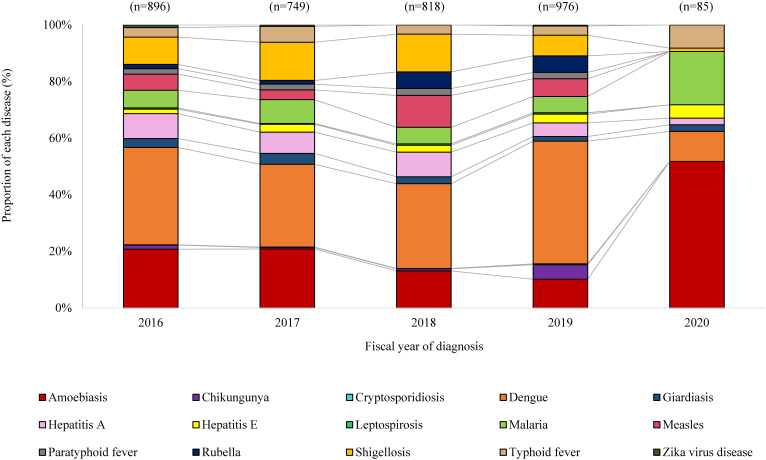
Proportionate distribution of imported infectious disease cases by year of diagnosis, Japan, April 2016–March 2021 (restricted to pre-selected 15 priority notifiable diseases)

Among the 15 imported infectious diseases, dengue accounted for the highest proportion in FY 2016–2019, averaging 34.7% (308/896 [34.4%], 219/749 [29.2%], 245/818 [30.0%] and 423/976 [43.3%] in FY 2016, 2017, 2018, and 2019, respectively) (Figure 3). However, the proportion of dengue declined to 10.6% (9/85) in FY 2020. Meanwhile, the proportion of amebiasis cases among the 15 diseases increased substantially from its average of 15.9% in FY 2016–2019 (186/896 [20.8%], 156/749 [20.8%], 107/818 [13.1%], and 99/976 [10.1%] in FY 2016, 2017, 2018, and 2019, respectively) to 51.8% (44/85) in FY 2020. Similarly, the proportion of malaria increased almost three-fold from 6.5% (56/896 [6.3%], 63/749 [8.4%], 48/818 [5.9%], and 56/976 [5.7%] in FY 2016, 2017, 2018, and 2019, respectively) to 18.8% (16/85) in FY 2020.

In all 15 diseases, the number of cases declined (Table 2). The largest decrease was observed in dengue, followed by amebiasis and shigellosis. However, none of the 15 diseases showed a substantial decline in the number of cases when accounting for the number of travelers; in fact, the number of dengue cases per million arrivals increased (Table 2). Relative to the pre-pandemic period, a two-fold or greater increase in the number of cases per arrival was observed for dengue, malaria, amebiasis, giardiasis, hepatitis A, hepatitis E, and typhoid fever. The highest absolute increase was observed in amebiasis, malaria, and typhoid fever, with an increase of 60.1 (95% CI, 41.5–78.7), 21.7 (95% CI, 10.5–33.0), and 9.3 (95% CI, 1.9–16.8) per million arrivals, respectively. Despite having the same modes of transmission,^12^ different trends were observed in the change in the number of cases per arrival for vector-borne (eg, malaria vs dengue) and food-borne infectious diseases (eg, typhoid fever vs paratyphoid fever).

**Table 2.** Numbers of imported infectious disease cases, imported infectious disease cases per 1,000,000 arrivals, and their ratios and differences during the pandemic compared to the pre-pandemic period, by disease, Japan, April 2016–March 2021

Main mode of transmission	Disease	Period^a^	Annual number of cases^b^	Ratio of the number of cases during the pandemic to the pre- pandemic period	Difference in the number of cases from the pre- pandemic period	Number of cases per million arrivals^c^	Ratio of the number of cases per million arrivals during the pandemic to the pre- pandemic period (95% CI)	Difference in the number of cases per million arrivals from the pre-pandemic period (95% CI)
Vector-borne (mosquito-borne)	Chikungunya	pre-pandemic	18	ref.	ref.	0.4	ref.	ref.
		pandemic	0	0.0	−18	0.0	0.0 (0.0–13.7)	−0.4 (−0.5 to −0.3)
	Dengue	pre-pandemic	299	ref.	ref.	6.5	ref.	ref.
		pandemic	9	0.0	−290	12.9	2.0 (0.9–3.8)	6.4 (−2.0 to 14.8)
	Malaria	pre-pandemic	56	ref.	ref.	1.2	ref.	ref.
		pandemic	16	0.3	−40	22.9	18.9 (10.6–31.4)	21.7 (10.5–33.0)
	Zika virus infection	pre-pandemic	4	ref.	ref.	0.1	ref.	ref.
		pandemic	0	0.0	−4	0.0	0.0 (0.0–63.9)	−0.1 (−0.1 to 0.0)
Food-borne/ water-borne	Amebiasis	pre-pandemic	137	ref.	ref.	3.0	ref.	ref.
		pandemic	44	0.3	−93	63.1	21.2 (15.2–28.8)	60.1 (41.5–78.7)
	Cryptosporidiosis	pre-pandemic	2	ref.	ref.	0.0	ref.	ref.
		pandemic	0	0.0	−2	0.0	0.0 (0.0–224.1)	0.0 (−0.1 to 0.0)
	Giardiasis	pre-pandemic	23	ref.	ref.	0.5	ref.	ref.
		pandemic	2	0.1	−21	2.9	5.7 (0.7–21.1)	2.4 (−1.6 to 6.3)
	Hepatitis A	pre-pandemic	63	ref.	ref.	1.4	ref.	ref.
		pandemic	2	0.0	−61	2.9	2.1 (0.3–7.6)	1.5 (−2.5 to 5.5)
	Hepatitis E	pre-pandemic	21	ref.	ref.	0.5	ref.	ref.
		pandemic	4	0.2	−17	5.7	12.4 (3.3–33.0)	5.3 (−0.3 to 10.9)
	Paratyphoid fever	pre-pandemic	19	ref.	ref.	0.4	ref.	ref.
		pandemic	0	0.0	−19	0.0	0.0 (0.0–13.5)	−0.4 (−0.5 to −0.3)
	Shigellosis	pre-pandemic	92	ref.	ref.	2.0	ref.	ref.
		pandemic	1	0.0	−91	1.4	0.7 (0.0–4.0)	−0.6 (−3.4 to 2.3)
	Typhoid fever	pre-pandemic	33	ref.	ref.	0.7	ref.	ref.
		pandemic	7	0.2	−26	10.0	14.1 (5.6–29.9)	9.3 (1.9–16.8)
Air-borne/droplet	Measles	pre-pandemic	58	ref.	ref.	1.2	ref.	ref.
		pandemic	0	0.0	−58	0.0	0.0 (0.0–0.0)	−1.2 (−1.4 to −1.1)
Droplet	Rubella	pre-pandemic	32	ref.	ref.	0.7	ref.	ref.
		pandemic	0	0.0	−32	0.0	0.0 (0.0–7.7)	−0.7 (−0.8 to −0.6)
Zoonosis	Leptospirosis	pre-pandemic	4	ref.	ref.	0.1	ref.	ref.
		pandemic	0	0.0	−4	0.0	0.0 (0.0–73.6)	−0.1 (−0.1 to 0.0)

CI, confidence interval.

^a^“Pre-pandemic” represents the period April 2016–March 2020, and “pandemic” represents the period April 2020–March 2021; both are based on the time of disease diagnosis.

^b^For the “pre-pandemic” period, the average annual number of cases for the period April 2016–March 2020 is presented.

^c^184,042,453 and 697,618 were used as the aggregate number of arrivals before and during the pandemic, respectively.

### Amebiasis case analysis

The proportion of male cases averaged 88% for amebiasis cases in FY 2016–2019 (163/186 [88%], 142/156 [91%], 92/107 [86%], and 85/99 [86%] in FY 2016, 2017, 2018, and 2019, respectively) and 86% (38/44) in FY 2020, showing no major changes before and during the pandemic. The proportion of asymptomatic cases increased from an average of 25.9% during FY 2016–2019 (42/186 [22.6%], 39/156 [25.0%], 32/107 [29.9%], and 26/99 [26.3%] in FY 2016, 2017, 2018, and 2019, respectively) to 34.1% (15/44) in FY 2020. The annual proportion of cases with unknown time of infection also increased from an average of 61.1% (120/186 [64.5%], 104/156 [66.7%], 65/107 [60.7%], and 52/99 [52.5%] in FY 2016, 2017, 2018, and 2019, respectively) to 70.5% (31/44) in FY 2020, as did the proportion with presumed infection for more than one year before diagnosis, from 7.5% (10/186 [5.4%], 11/156 [7.1%], 10/107 [9.3%], and 8/99 [8.1%], respectively) to 15.9% (7/44).

### Malaria case analysis

The proportion of non-Japanese malaria cases increased to 68.8% (11/16) during the pandemic, compared to an average annual proportion of 55.3% in FY 2016–2019 (28/56 [50.0%], 44/63 [69.8%], 23/48 [47.9%], and 30/56 [53.6%] in FY 2016, 2017, 2018, and 2019, respectively) before the pandemic. The absolute increase in non-Japanese notifications per million foreign national arrivals was 31.1 (95% CI, 12.0–50.1) (Table 3), higher than 21.7 (95% CI, 10.5–33.0) when including all arrivals. When stratified by region, excluding five cases (pre-pandemic, 4/125; pandemic, 1/11) with unknown or multiple suspected countries of infection, the majority of non-Japanese cases consistently originated from Africa (38/56 [67.9%], 47/63 [74.6%], 38/48 [79.2%], 45/56 [80.4%], and 12/16 [75.0%] in FY 2016, 2017, 2018, and 2019, respectively). Following the pandemic, the absolute increase in non-Japanese cases per million foreign national arrivals from Africa was high, at 1,537.7 (95% CI, 88.9–2,986.6). In terms of the relative increase, the number of non-Japanese cases per foreign arrivals from all regions pooled and Africa rose 28-fold and four-fold, respectively (Table 3).

**Table 3.** Numbers of non-Japanese imported malaria cases, non-Japanese imported malaria cases per 1,000,000 foreign national arrivals, and their ratios and differences during the pandemic compared to the pre-pandemic period, by region, Japan, April 2016–March 2021

			Japanese and non-Japanese	Non-Japanese					

Origin of travel		Period^a^	Annual number of cases^b^	Annual number of cases^b^	Ratio of the number of cases during the pandemic to the pre- pandemic period	Difference in the number of cases from the pre- pandemic period	Number of cases per million arrivals^c^	Ratio of the number of cases per million arrivals during the pandemic to the pre-pandemic period (95% CI)	Difference in the number of cases per million arrivals from the pre-pandemic period (95% CI)
All regions		pre-pandemic	56	31	ref.	ref.	1.1	ref.	ref.
		pandemic	16	11	0.4	−20	32.2	28.4 (13.8–52.6)	31.1 (12.0–50.1)
By region									
	Africa	pre-pandemic	42	25	ref.	ref.	547.2	ref.	ref.
		pandemic	12	8	0.3	−17	2,085.0	3.8 (1.6–7.8)	1,537.7 (88.9–2,986.6)
	Asia	pre-pandemic	8	6	ref.	ref.	0.2	ref.	ref.
		pandemic	2	2	0.3	−4	7.3	29.3 (3.3–118.5)	7.0 (−3.1 to 17.1)
	Europe	pre-pandemic	0	0	ref.	ref.	0.0	ref.	ref.
		pandemic	0	0	N/A	0	0.0	N/A	0.0 (0.0–0.0)
	North America	pre-pandemic	0	0	ref.	ref.	0.0	ref.	ref.
		pandemic	0	0	N/A	0	0.0	N/A	0.0 (0.0–0.0)
	Oceania	pre-pandemic	2	0	ref.	ref.	0.0	ref.	ref.
		pandemic	0	0	N/A	0	0.0	N/A	0.0 (0.0–0.0)
	South America	pre-pandemic	0	0	ref.	ref.	0.0	ref.	ref.
		pandemic	0	0	N/A	0	0.0	N/A	0.0 (0.0–0.0)

CI, confidence interval; N/A, not applicable.

^a^“Pre-pandemic” represents the period April 2016–March 2020, and “pandemic” represents the period April 2020–March 2021; both are based on the time of disease diagnosis.

^b^For the “pre-pandemic” period, the average annual number of cases for the period April 2016–March 2020 is presented.

^c^110,237,475 and 341,479 were used for the analysis in all regions as the aggregate number of foreign nationals arriving in Japan before and during the pandemic, respectively. For regional analysis, the following respective numbers were used: 179,082 and 3,837 for Africa; 92,545,213 and 274,896 for Asia; 6,961,301 and 19,140 for Europe; 1,877,022 and 13,332 for North America; 2,432,713 and 2,164 for Oceania; and 607,341 and 10,343 for South America.

## DISCUSSION

The COVID-19 pandemic saw a notable decrease in traveler volume to Japan and a drastic shift in their characteristics, suggesting that the travel situation was considerably affected by travel restrictions and other related measures. Notably, the annual number of all 15 imported infectious disease cases decreased, along with a marked decline in the number of arrivals.^4^ This was consistent with pre-pandemic findings, which showed a positive correlation between the passenger volume and the number of imported cases.^7^ However, this decline differed by disease and the proportion of malaria cases increased, indicating a proportionately greater importance of malaria importation for Japan.

Moreover, seven of the 15 diseases showed two-fold or greater relative increase in the number of cases per arrival during versus before the pandemic, implying that the relative “risk” of detecting the disease among arrivals actually increased (though strictly speaking, “risk” represents a crude ratio of the number of cases to that of arrivals in a given time period, and the denominator may have included those not considered to be at risk for some of the diseases). Furthermore, while dengue showed a large decrease in both the case counts and as a proportion among the 15 diseases, the notification rate among travelers had doubled. Notably, in addition to the relative increase, the substantial absolute increase accounting for travelers was observed for amebiasis, malaria, and typhoid fever. Therefore, despite the decrease in the number of importations, the relative and absolute risk among travelers for several diseases showed an appreciable increase.

The ratio comparing the notification rate accounting for arrivals between the two periods indicates a relative change. As with the concept of risk difference,^13^ on the other hand, considering the difference in notifications per arrival accounts for the absolute risk and can quantify the notification rate change in absolute terms. Its importance can be illustrated by an example among non-Japanese malaria importations. The ratio of the number of cases per million arrivals during the pandemic compared to that of the pre-pandemic period was 28 for all regions (pooled) versus 4 for Africa. This suggests that the pandemic period increased the risk of malaria importation relatively more among those from all regions compared to those from Africa. However, when the difference was considered, the order was reversed, being 31 and 1,538 per million foreign national arrivals, respectively. Hence, during the pandemic, while the malaria notification rate showed a greater relative increase for all regions (pooled), the absolute risk of malaria importation increased more among those from Africa. Similarly, while the notification rate for giardiasis increased six-fold, while that for dengue only doubled, the absolute change in the notification rate per million arrivals was 2 for giardiasis and 6 for dengue. Thus, information provided by the difference in case counts per arrival can also be useful for public health decision making.^13^

Given the ongoing high risks among travelers, the following pre-pandemic travel-related concerns may be present: first, some of these diseases including malaria and dengue may cause fatal outcomes, especially in high-risk travelers^14^^,^^15^; second, physicians in non-endemic countries are unfamiliar with certain infectious diseases and are less likely to include them for differential diagnosis, which may delay diagnosis and treatment. In addition to these previously highlighted issues, new concerns have been raised under the pandemic: co-infection with dengue and COVID-19 has been reported to be associated with severe and fatal outcomes^16^; physicians may overlook some infectious diseases by focusing on COVID-19^17^; there may also have been changes in healthcare-seeking behavior and challenges in accessing healthcare. Therefore, considering the continued high risk among travelers, despite the decline in notified case counts, public health authorities should continue their efforts to ensure that patients receive early diagnosis and treatment to prevent serious outcomes. Such quantitative evaluation of the risks posed to travelers could help public health practitioners to effectively communicate with physicians, improving their awareness.

In the additional analysis of amebiasis, the proportion of asymptomatic cases that may have been diagnosed incidentally,^11^ cases that took more than 1 year from presumed infection to diagnosis, and cases with an unknown time of infection increased. These results suggest that cases diagnosed during the pandemic involved a certain number of those infected before the pandemic and the data may not reflect the trend of amebiasis imported during the pandemic. Therefore, given the drastic decrease in the number of travelers, the case counts among arrivals could have resulted in an apparent increase.

For malaria, the following factors may have contributed to their increased notifications per arrival. The largest number of malaria cases were reported as infected in Africa. In 2020, Sub-Saharan Africa experienced a malaria epidemic,^17^ which may have contributed to the absolute increase in the notification rate among all arrivals. The difference in the number of cases per arrival was found to be larger when restricted to non-Japanese than when including non-Japanese and Japanese. This indicates that the absolute risk greatly increased among the non-Japanese. Particularly, when stratified by region, the increase among African national arrivals was larger than that among those from other regions. Regarding the characteristics of foreign national arrivals, the proportion of temporary visitors and those with non-working status decreased substantially during the pandemic. In contrast, the distribution of those with employment or residence visas had increased considerably. Given this context and known risk factors for malaria among travelers, such as visiting friends and relatives (VFRs), long-term stay, and travel to endemic countries,^18^^,^^19^ the proportion of high-risk arrivals including long-term residence in endemic areas and VFR returnees may have increased. This may have contributed to the substantial increase in notifications among African arrivals. In addition, it is possible that the increased proportion of foreign national travelers staying in Japan through the incubation period made them more likely to be detected domestically. Meanwhile, the proportion of African nationals among foreign national arrivals remained very low. Taken together, the risk of malaria among all travelers entering Japan could have been affected by travelers’ characteristics and local epidemics rather than by change in the travel volume from Africa.

Based on these findings, we believed that the altered travel situation, such as the demographic composition, length of stay, and destination/origin of travel, may have affected the trends and distributions of imported infectious diseases per travelers in FY 2020, although there may be variations in degree depending on individual diseases. Due to the implementation of strict border control measures throughout the world during the same period, such findings (eg, malaria) may also have been observed in other countries and be of relevance. As the COVID-19 pandemic shifts towards an endemic phase, many countries, including Japan, are further relaxing travel restrictions. With such a drastic change in the travel context, not only describing trends in the number of cases but accounting for the number of travelers would be useful for public health authorities to assess risk for response. Furthermore, considering the difference in the notification rate among travelers can inform them about meaningful absolute changes, contributing to their selection of diseases that should be prioritized for action, particularly when resources are overwhelmed by public health emergencies such as COVID-19.

Our study has several limitations. First, trends in distributions may not be captured to the same extent as that before the pandemic if health-seeking behaviors or testing capacities had changed. If the implementation of health monitoring for arrivals during their quarantine period^20^ had facilitated detection of individuals with imported diseases other than COVID-19, case detection during the pandemic may have become more sensitive. Second, overestimation may have also occurred for diseases that can take a long time from infection to diagnosis, such as amebiasis. Third, non-Japanese cases of malaria may have been misclassified by assumptions based on their names. However, the misclassification was expected to have occurred equally before and during the pandemic and this would have had little impact. Fourth, in the analysis of the number of cases per arrival by region, the numerator was the number of cases by region of infection and the denominator was the number of travelers by nationality; thus, a proportion of the numerator may not have been included in the denominator.

### Conclusions

Although the number of cases notified under the national infectious disease surveillance scheme decreased for all 15 imported infectious diseases during the COVID-19 pandemic in Japan, relative increase in cases per travelers was observed for several diseases. Moreover, the number of cases per million travelers increased considerably for amebiasis, malaria, and typhoid fever. In the context of drastic shifts in travel patterns, it is essential to account for the number of travelers and consider changes in both relative and absolute terms. Our findings and these considerations are important for public health practitioners to communicate to physicians to facilitate vigilance against imported infectious diseases.

## SUPPLEMENTARY MATERIAL

eFigure 1. Demographic and travel statistics of Japanese national arrivals, 2016–2021

eFigure 2. Demographic and travel statistics of foreign national arrivals, 2016–2021
