# Supplementary material for: Epidemiologic Trends and Distributions of Imported Infectious Diseases Among Travelers to Japan Before and During the COVID-19 Pandemic, 2016 to 2021: A Descriptive Study
Source: J Epidemiol. 2024 Apr 5;34(4):187–94. doi: 10.2188/jea.JE20230025 (PMC10918336; doi:10.2188/jea.JE20230025)

eFigure 1. Demographic and travel statistics of Japanese national arrivals, 2016–2021

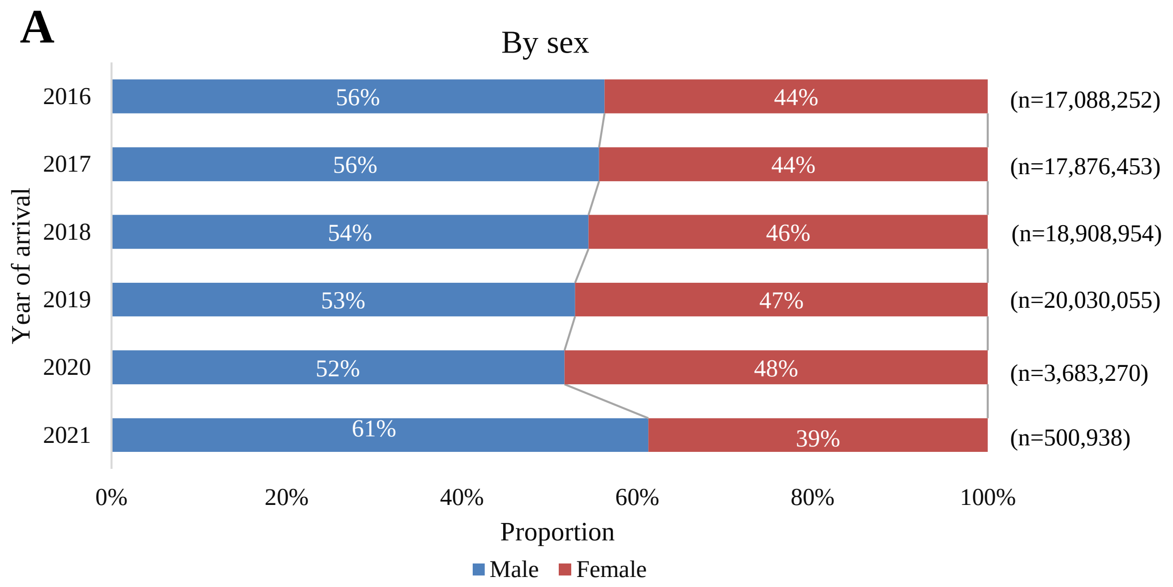

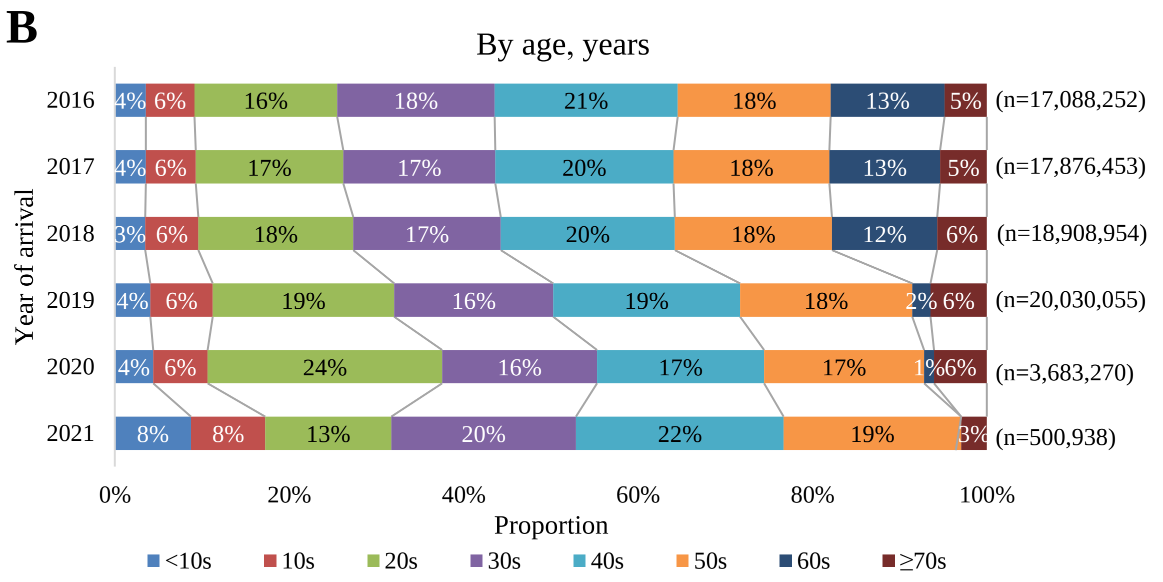

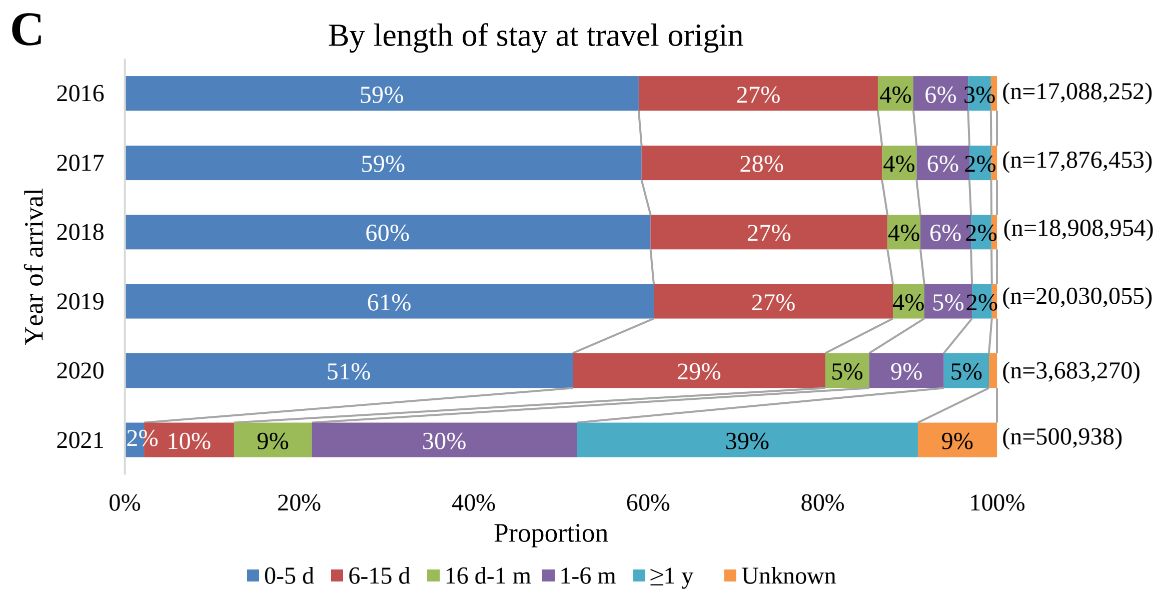

Due to limitations in the available data, the figures are not presented by fiscal year but by calendar year starting in January. Therefore, the characteristics of the arrivals in the calendar year 2020 reflect those around January–March 2020 (FY2019), when relatively higher volumes of travelers were observed.

eFigure 2. Demographic and travel statistics of foreign national arrivals, 2016–2021

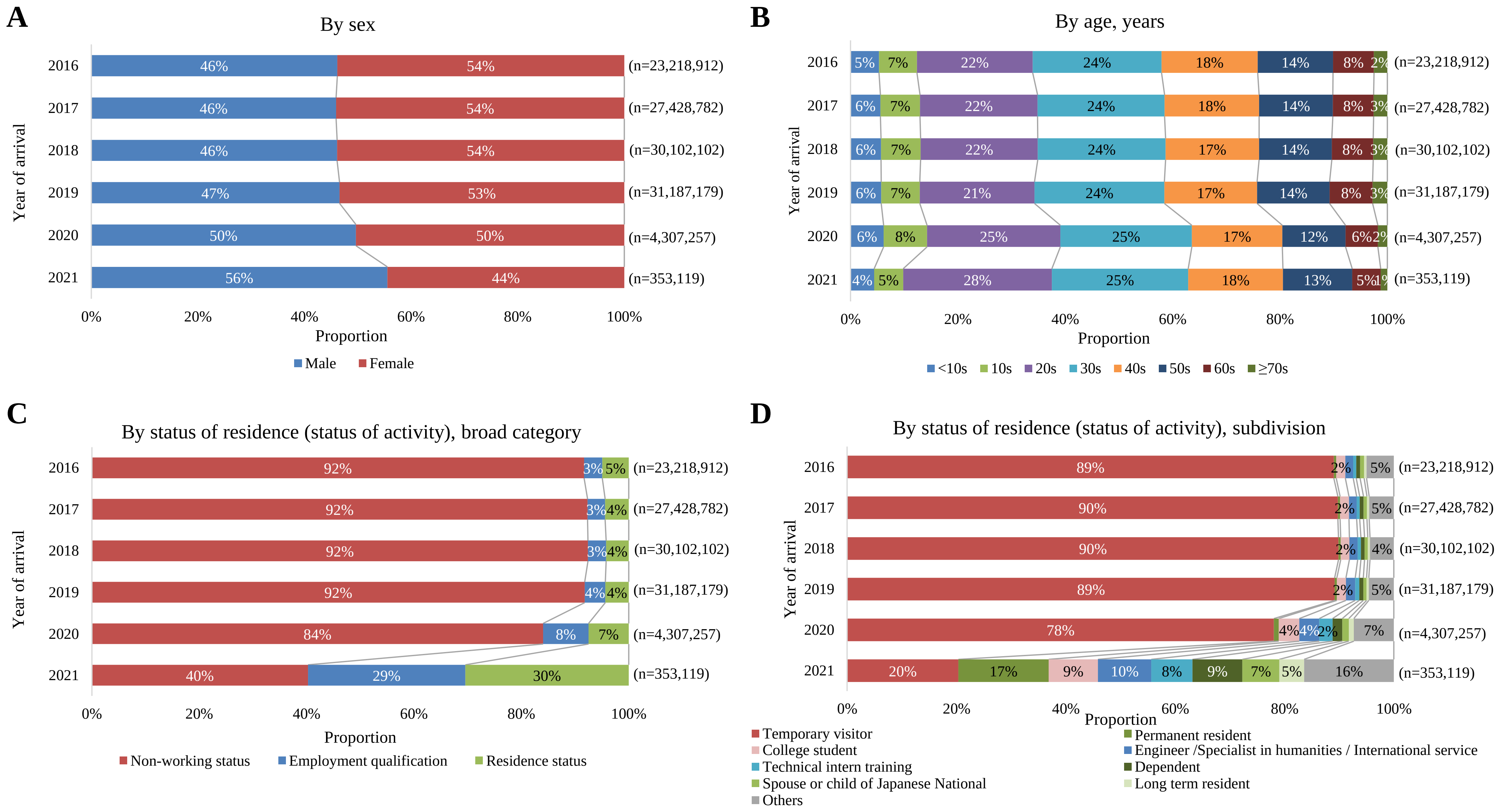

Supplement: Supplementary file 1 [file je-34-187-s001.pdf]
